# Descending Necrotizing Mediastinitis: A Rare Yet Lethal Complication Demanding Timely Intervention

**DOI:** 10.7759/cureus.76459

**Published:** 2024-12-27

**Authors:** Anastasia Kosmidou, Panagiota Kosmidou, Ioannis Karamatzanis, Eleni Flari, Sofia Pouriki, Panagiotis Κ Nikolopoulos

**Affiliations:** 1 Pulmonology, Sotiria General Hospital of Thoracic Diseases, Athens, GRC; 2 Otolaryngology-Head and Neck Surgery, University of Patras Medical School, Patras, GRC; 3 Otolaryngology-Head and Neck Surgery, German Medical Institute of Cyprus, Limassol, CYP; 4 Internal Medicine, Basildon University Hospital, Basildon, GBR; 5 Biomedical Sciences, College of Medicine and Veterinary Medicine, Edinburgh Medical School, University of Edinburgh, Edinburgh, GBR; 6 Dentistry and Biomedical Sciences, Queen's University Belfast School of Medicine, Belfast, GBR; 7 Intensive Care Unit, General Chest Diseases Hospital Sotiria, Athens, GRC

**Keywords:** descending mediastinitis, descending necrotizing mediastinitis, ent pathology, mediastinitis management, multidisciplinary management

## Abstract

Descending necrotizing mediastinitis (DNM) is a rare and potentially life-threatening condition characterized by the rapid spread of infection within the mediastinum. This severe form of mediastinitis poses a significant challenge to clinicians due to its aggressive nature and potential for rapid deterioration. In this case report, we present a challenging case of descending necrotizing mediastinitis in a 39-year-old patient with persistent pyrexia and an extended hospital stay in the intensive care unit (ICU), cardiothoracic unit (CTU), and surgical intensive care unit (SICU). The step-by-step clinical course, diagnostic challenges, and therapeutic strategies will be discussed in detail. This case highlights the importance of early recognition and a comprehensive, interdisciplinary approach in managing this rare yet lethal mediastinitis.

## Introduction

Descending necrotizing mediastinitis (DNM) is a rare and life-threatening condition characterized by the spread of infection from head and neck infections to the mediastinum [1]. This severe form of mediastinitis poses significant challenges to clinicians due to its aggressive nature and potential for rapid deterioration. First described by Pearse in 1938, DNM remains a rare yet severe condition, with a mortality rate ranging from 20% to 40% [2]. Recent advancements in imaging modalities and antibiotic therapies have helped lower this statistic [2,3]; however, the disease remains a significant concern. Notably, delayed diagnosis and rapid progression frequently result in sepsis and multiorgan failure [1].

The condition typically arises from oropharyngeal (including tonsillar and dental), retropharyngeal, and cervical infections [4]. The anatomical proximity of these structures to the mediastinum allows for a downward spread of disease along the superficial and deep fascial planes of the neck, most commonly into the posterior mediastinum, where a plethora of thoracic nerves, vasculature, and organs are located [4]. The typical presentation includes chest pain, pyrexia, neck or chest swelling, and respiratory distress [3]. Risk factors linked to the development of DNM include smoking, diabetes, renal failure, obesity, and an immunocompromised state [3].

The diagnostic criteria for DNM were first established by Estrera et al. in 1983 and include 1) clinical manifestations of severe infection, 2) characteristic radiographic findings, 3) the documentation of necrotizing mediastinal infection in operation, and 4) the establishment of a primary oropharyngeal or cervical infection with DNM progression [5]. Clinical signs associated with cervical involvement include pain, difficulty swallowing, loss of appetite, shortness of breath, fever, painful swallowing, and anterior neck swelling [4]. Radiological features from computed tomography (CT) are used to determine the extent of mediastinal involvement and provide a baseline for postoperative comparison [5]. Finally, as described, the intraoperative evidence of infection and the identification of an original focus of infection are required to make a diagnosis of DNM.

Moreover, DNM requires a high index of clinical suspicion and a multidisciplinary approach for effective management, as the diagnosis is often delayed due to its nonspecific infectious symptoms [1]. While a chest X-ray may reveal signs such as tracheal displacement from cervical swelling, widened mediastinum, and pleural effusion [6], the gold standard imaging for confirming DNM is contrast-enhanced cervical-thoracic computed tomography (CT) [3]. Diagnostic CT features described by Scaglione et al. include fluid collections with or without gas in the mediastinum, pleura, or pericardium; increased cervical fat or cellulitis; myositis; lymphadenopathy; and vascular thrombosis [7]. Immediate and aggressive intervention, typically involving surgical drainage and the administration of broad-spectrum antimicrobial therapy, is crucial to prevent disease progression and improve outcomes. Upon diagnosis, the patient should be transferred to the intensive care unit (ICU) for close monitoring.

In this case report, we present a challenging case of descending necrotizing mediastinitis in a 39-year-old man with persistent pyrexia. The clinical course, diagnostic challenges, and therapeutic strategies are discussed in detail. The case underscores the critical importance of the early recognition of DNM, emphasizing the need for a comprehensive, interdisciplinary approach to managing this rare yet lethal form of mediastinitis. With this article, we aim to raise awareness of this life-threatening condition resulting from common head and neck infections and to encourage early recognition and prompt management.

## Case presentation

A 39-year-old Greek man presented to the emergency department of Sotiria Thoracic Diseases Hospital in Athens with symptoms of dysphagia, retrosternal chest pain, and dyspnea. The patient reported a three-day history of pyrexia reaching 40°C, accompanied by headache, generalized weakness, and fatigue. The patient reported severe toothache for five days prior to presentation with associated pus production. The patient had no significant past medical history, was not taking any regular medications, and had no known allergies. He had a 20-pack-year smoking history and was a social drinker.

**Figure 1:**
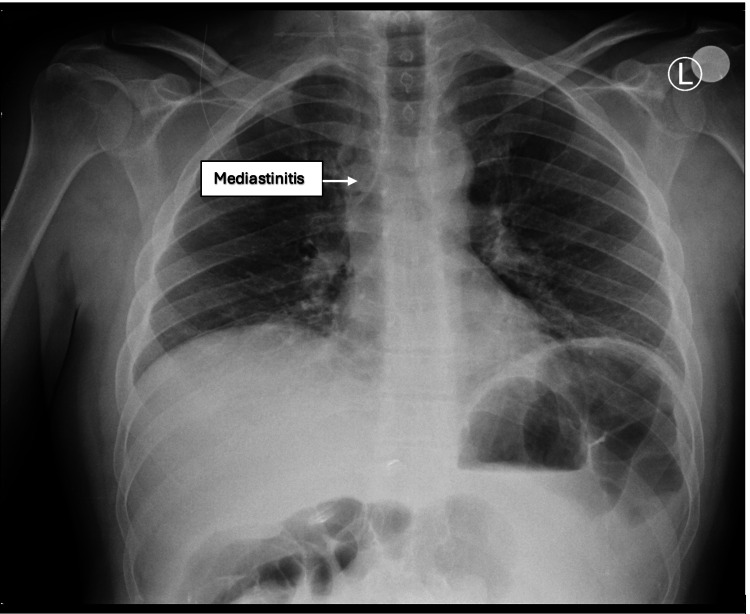
The patient's chest X-ray on admission. The presence of the widening and haziness of the mediastinum as indicated by the white arrow prompted further investigations.

On initial examination, the patient appeared unwell yet stable. The vital signs were reported as a temperature of 38.4°C, a saturation of peripheral oxygen (SatO_2_) of 93% on room air, a heart rate (HR) of 135 beats per minute (bpm), and a blood pressure (BP)of 105/69 mmHg (National Early Warning Score 2 {NEWS2} score of 7). Auscultation revealed bilateral crackles at the lung bases. Notably, there was swelling in the right submandibular area, with erythema, warmth, and a palpable crepitus. The electrocardiogram (ECG) showed sinus tachycardia. The initial tests ordered included a chest X-ray (Figure 1) and blood tests (Table 1).

**Table 1:** Blood results on day 1. Several abnormalities were noted, including high WBC count, abnormal clotting times, raised inflammatory markers, and deranged LFTs. WBC, white blood cells; NEU, neutrophils; LYMPH, lymphocytes; HGB, hemoglobin; HCT, hematocrit; MCV, mean corpuscular volume; MCH, mean corpuscular hemoglobin; MCHC, mean corpuscular hemoglobin concentration; PLT, platelets; INR, international normalized ratio; PT, prothrombin time; PTT, partial thromboplastin time; CRP, C-reactive protein; ESR, erythrocyte sedimentation rate; U&E, urea and electrolyte; CREA, creatinine; Na, sodium; K, potassium; LFT, liver function test; ALB, albumin; ALP, alkaline phosphatase; T.Bil, total bilirubin; GGT, gamma-glutamyl transferase; ALT, alanine transaminase; AST, aspartate transferase; LDH, lactate dehydrogenase; GLU, glucose; CPK, creatine phosphokinase

FBC			Clotting			Inflammatory Markers		
WBC (×10^9^/L)	15.7	4.0-11.0	INR	1.35	0.8-1.2	CRP (mg/L)	33	<2
NEU (%)	90	20-75	PT (seconds)	13.2	7.6-10.6	ESR (mm/hour)	82	<10
LYMPH (%)	1.7	10-45	PTT (seconds)	36.3	26.0-38.0			
ΗGB (g/L)	146	134-167						
HCT (%)	44.8	39.2-48.6						
MCV (fL)	87.3	79.6-94.0						
MCH (pg/cell)	28.5	27.3-32.8						
MCHC (g/L)	326	324-363						
PLT (×10^9^/L)	269	150-410						
U&E			LFT			Others		
Urea (mmol/L)	5.6	2.5-7.8	ALB (g/dL)	3.4	3.5-5.2	GLU (mg/dL)	119	<200
CREA (μmol/L)	130	70-150	ALP (U/L)	67	45-115	CPK (mcg/L)	687	10-120
Na (mmol/L)	134	135-145	T.Bil (mg/dL)	0.9	0.2-1.3			
K (mmol/L)	4.3	3.5-5.0	GGT (U/L)	105	11-51			
			ALT (U/L)	66	7-56			
			AST (U/L)	56	8-45			
			LDH (U/L)	267	122-222			

**Figure 2:**
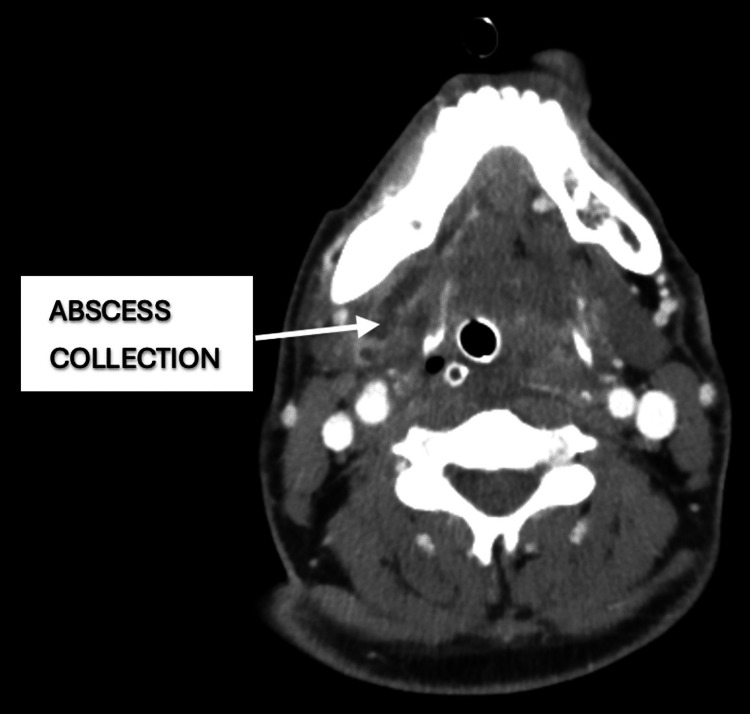
CT of the neck showing the 4.5 cm abscess in the submandibular-submental space. CT: computed tomography

A contrast-enhanced computed tomography pulmonary angiography (CTPA) was performed subsequently, which excluded the differential diagnosis of pulmonary embolism, and revealed the presence of pneumomediastinum. The scan also identified an "opacification of the subcutaneous fat tissue in the area of the sternal handle." A CT of the neck area (Figure 2) found a 4.5 cm abscess in the submandibular-submental space and bilateral lymph node enlargement in both jugular spaces. Furthermore, there was evidence of free air in the submandibular, parapharyngeal, jugular, and prevertebral spaces, which extended into the superior mediastinum.

The abscess was initially treated conservatively with admission to the ENT department for intravenous (IV) antibiotics, analgesia, and gastroprotection with intravenous omeprazole (Table 2).

**Table 2:** Medication administered upon admission to the ENT unit (day 1). IV, intravenous; TDS, three times a day; BD, twice a day; OD, once a day

Medication and Route	Dose	Frequency
IV ceftazidime	2 g	TDS
IV metronidazole	500 mg	TDS
IV amikacin	500 mg	BD
IV tramadol	100 mg	TDS
IV omeprazole	40 mg	OD

Due to the presence of air in the mediastinum, the patient was referred to the cardiothoracic unit (CTU). At the time of assessment, pneumomediastinum was present, but there were no features suggestive of mediastinitis. The CTU recommended the continuation of triple IV antibiotics, the initiation of mechanical ventilation at 35% oxygen, a new neck-chest CT in three days, and reassessment in case of clinical deterioration.

On the same day, there was evident clinical deterioration. Bedside observations revealed a temperature of 41°C, oxygen saturation of 93% despite mechanical ventilation, an HR of 109 bpm, and a BP of 95/45 mmHg (NEWS-2 score of 11). These findings, in conjunction with the CT results, led to the decision for the patient to be urgently transferred for a right thoracotomy, in view of his hemodynamic instability. During the surgery, part of the pericardial pleura was removed, partial lung exfoliation was performed, and two chest drains were inserted on the right side. Post-surgery, a purulent collection was discovered in the hemithorax, along with areas of necrosis in the posterior mediastinal pleura. Consequently, the decision was made to intubate the patient and admit them to the surgical intensive care unit (SICU).

**Figure 3:**
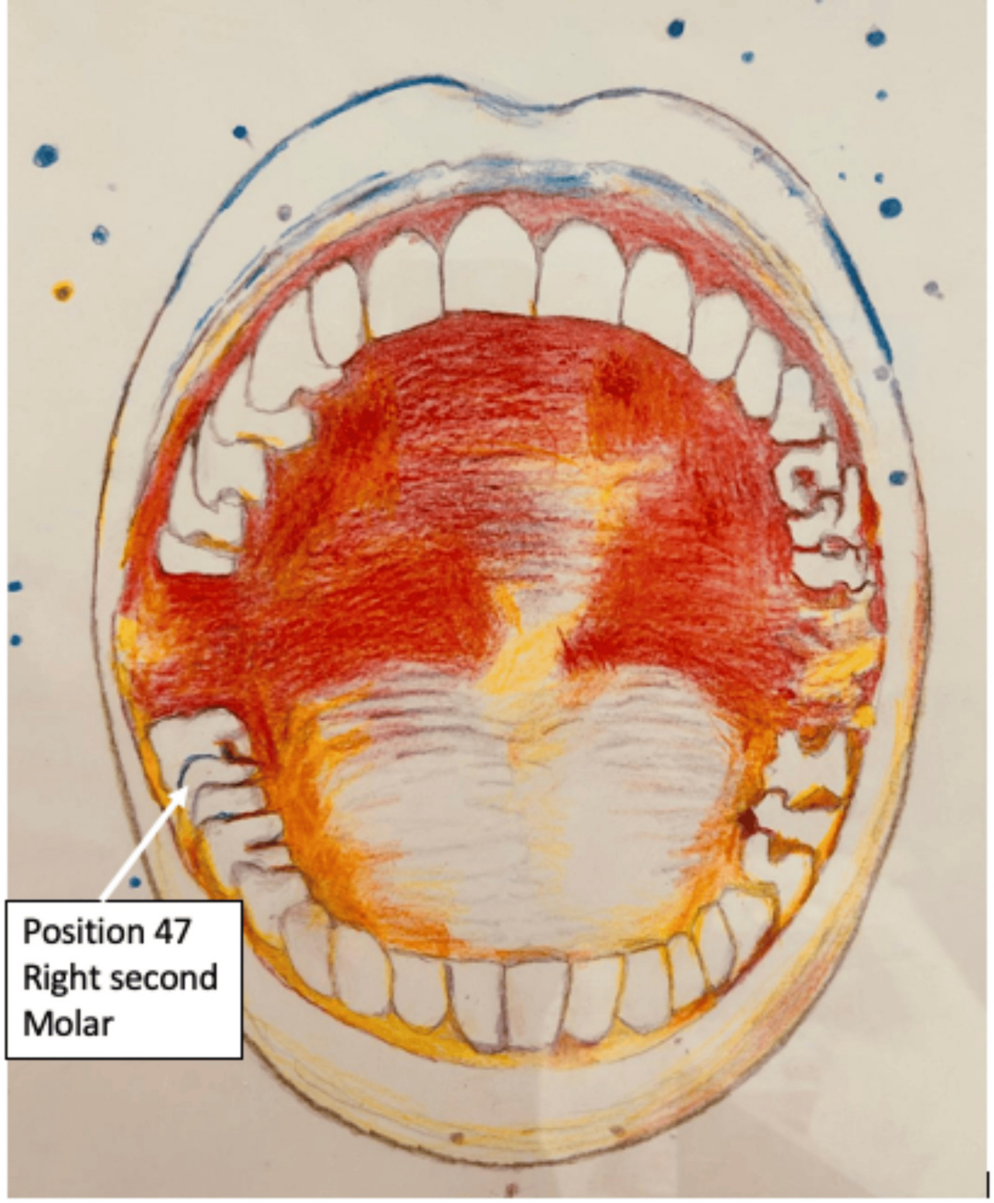
Illustration of the position of the extracted tooth. Image credit: Nikos Kosmidis

While in the SICU, the patient was referred for a maxillofacial surgery evaluation. The assessment revealed diffuse inflammation in the neck area and multiple decayed teeth in the oral cavity. The drainage of pus and the tooth extraction of the right second lower molar (position 47) were conducted (Figure 3). Abnormal blood test results from SICU admission can be found in Table 3.

**Table 3:** Abnormal blood results post-admission to the SICU. The WBC remains high, with deranged U&E and LFTs. WBC, white blood cells; NEU, neutrophils; CREA, creatinine; CRP, C-reactive protein; ALB, albumin; T.Bil, total bilirubin; GGT, gamma-glutamyl transferase; LDH, lactate dehydrogenase; AMY, amylase; CPK, creatine phosphokinase; ALP, alkaline phosphatase; AST, aspartate transferase; ALT, alanine transaminase; SICU, surgical intensive care unit; U&E, urea and electrolyte; LFT, liver function test

FBC			U&E			Inflammation Markers			LFTs		
WBC (×10^9^/L)	16.8	4.0-11.0	Urea (mmol/L)	10.3	2.5-7.8	CRP (mg/L)	8.9	<2	ALB (g/dL)	2.7	3.5-5.2
NEU (%)	88.8	20-75	CREA (μmol/L)	110	70-150				T.Bil (mg/dL)	1.3	0.2-1.3
									GGT (U/L)	265	11-51
									LDH (U/L)	613	122-222
									AMY (U/L)	508	30-110
									CPK (mcg/L)	409	10-120
									ALP (U/L)	67	45-115
									AST (U/L)	282	8-45
									ALT (U/L)	309	7-56

A microbiology consultation was requested due to the persistence of fever despite broad-spectrum antibiotic coverage (Table 4 shows the antibiotics and other medications administered while in the SICU). Microbiology advice included a) changing the antibiotic treatment to meropenem, b) exploring other potential inflammatory foci for drainage if the patient's condition did not improve, and c) conducting a triplex ultrasound of the jugular veins.

**Table 4:** Medications administered while in the SICU. IV, intravenous; TDS, three times a day; BD, twice a day; OD, once a day; SICU, surgical intensive care unit

Medication and Route	Dose	Frequency
IV metronidazole	500 mg	TDS
IV ciprofloxacin	500 mg	BD
IV piperacillin/tazobactam	100 mg	TDS
IV linezolid	600 mg	BD
IV omeprazole	40 mg	OD

**Figure 4:**
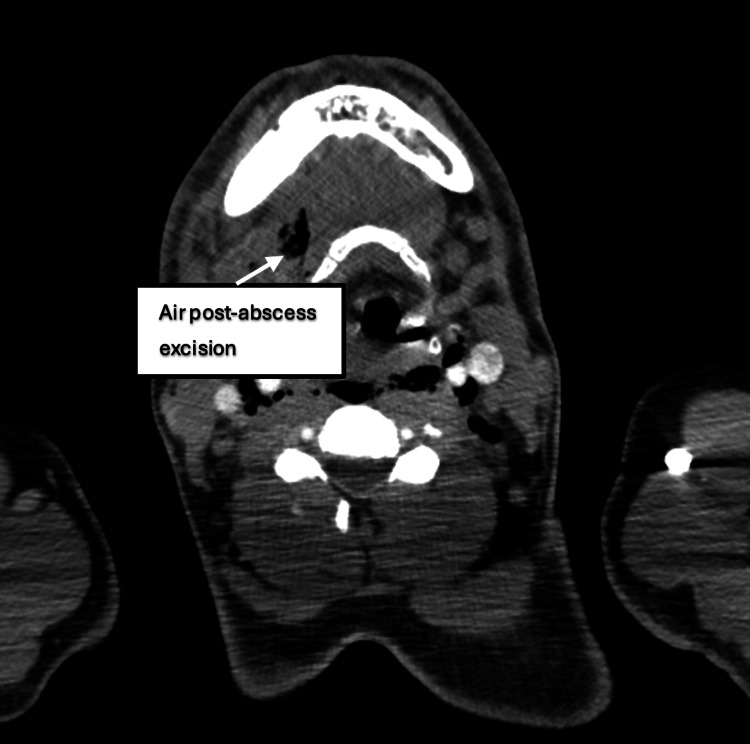
CT of the neck showing the presence of air following the excision of abscess (day 5). CT: computed tomography

In the following days, the patient was stable: a CT of the brain, neck (Figure 4), chest, and upper and lower abdomen was performed. Tiny air bubbles in the submental and submandibular spaces were seen in the cervical region, and edematous changes were observed in the right parapharyngeal space and cervical lymph nodes. In the chest, pneumomediastinum recession was seen. Abdominal CT revealed an extensive collection of fluid in the pelvis.

A subsequent maxillofacial assessment recommended the removal of the intra-oral drain based on teeth extraction. The patient was then transferred to the medical intensive care unit (MICU). Upon admission to the MICU, the diagnoses included a) dental infection, b) parapharyngeal abscess, c) descending necrotizing mediastinitis, and d) septic shock.

**Figure 5:**
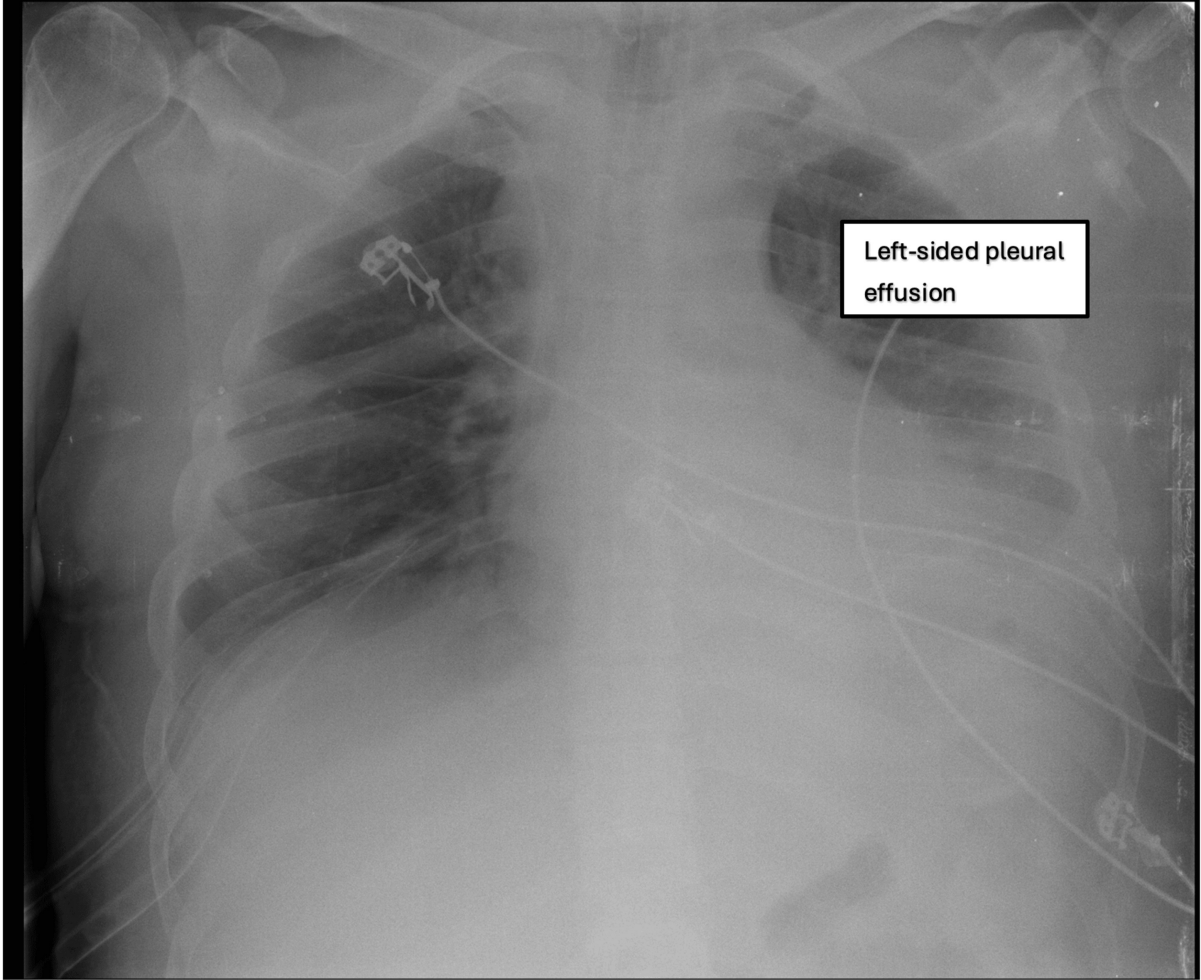
Chest X-ray showing new, left-sided pleural effusion on the day of transfer to the MICU (day 6 of hospitalization). MICU: medical intensive care unit

The multidisciplinary team's plan of action upon transfer was as follows: primarily, to manage septic shock, which included obtaining cultures from blood, urine, and bronchial secretions and administering the most appropriate antibiotic treatment based on the identified pathogen; next, to perform maxillofacial surgical intervention to address intra-oral pus and the exacerbation of neck swelling; and finally, to monitor clinical progress through serial chest X-rays (Figure 5) and blood tests (Table 5) and to review and amend medications as needed (Table 6). Specifically, meropenem and noradrenaline were added to address septic shock, while other medications were adjusted to manage the patient's pain and sedation levels.

**Table 5:** Abnormal blood results on day 6. The worsening of LFTs is observed. WBC, white blood cells; NEU, neutrophils; CREA, creatinine; CRP, C-reactive protein; ALB, albumin; T.Bil, total bilirubin; GGT, gamma-glutamyl transferase; LDH, lactate dehydrogenase; AMY, amylase; CPK, creatine phosphokinase; AST, aspartate transferase; ALT, alanine transaminase; U&E, urea and electrolyte; LFT, liver function test; PCT, procalcitonin; INR, international normalized ratio; PT, prothrombin time; PTT, partial thromboplastin time; FBC, full blood count

U&E			LFT			FBC, Clotting Screen, and Others		
Urea (mmol/L)	4.1	2.5-7.8	ALB (g/dL)	2.7	3.5-5.2	WBC (×10^9^/L)	17.3	4.0-11.0
CREA (μmol/L)	88	70-150	AMY (U/L)	450	30-110	NEU (%)	90	20-75
			T.Bil (mg/dL)	1.8	0.2-1.3	CRP (mg/L)	14	<2
			GGT (U/L)	364	11-51	INR	1.73	0.8-1.2
			ALT (U/L)	316	7-56	PT (seconds)	22.3	7.6-10.6
			AST (U/L)	305	8-45	PTT (seconds)	34.1	26.0-38.0
			LDH (U/L)	650	122-222	CPK (mcg/L)	714	10-120
			PCT (ng/mL)	3.02	<0.05			

**Table 6:** Medication list on day 6. Broad-spectrum antibiotics (meropenem) and vasopressors (noradrenaline) was used to address septic shock. IV, intravenous; NaCl, sodium chloride; PRN, as needed; TDS, three times a day; BD, twice a day; OD, once a day; QDS, four times a day

Route and Medication	Dose	Frequency
IV metronidazole	600 mg	TDS
IV meropenem	2 g	TDS
IV linezolid	600 mg	BD
IV omeprazole	40 mg	BD
IV furosemide	10 mg	BD
IV noradrenaline	15 g	OD
IV 0.9% NaCl	1 L/24 hours	OD
IV paracetamol	1 g (PRN)	QDS

Further investigations were performed to monitor progress. An echocardiogram reported a left ventricular ejection fraction (LVEF) of 60% with no abnormal findings. CT of the head-neck-chest-upper and lower abdomen was performed to assess the progression of the disease, and an abscess of the submandibular region with extension to the submental space and the thyroid cartilage was reported. Moreover, we sought a reassessment from the maxillofacial surgery team in light of the above findings. The team performed abscess drainage on the right parapharyngeal, submental, and submandibular spaces, with a large discharge of pus and three external and one internal drain placement. Cultures sent from the right parapharyngeal and submandibular abscesses revealed the presence of *Klebsiella pneumoniae*. These abscesses were treated with gentamicin and ceftazidime. The chest drain cultures were negative. The patient's antibiotic regimen was changed according to the new findings (Table 7).

**Table 7:** Changes to antibiotic regimen following maxillofacial team assessment. IV, intravenous; TDS, three times a day; BD, twice a day; OD, once a day

Route and Medication	Dose	Frequency
IV metronidazole	500 mg	TDS
IV ceftazidime/avibactam	2.5 g	TDS
IV linezolid	600 mg	BD
IV gentamicin	480 mg	OD
IV micafungin	100 mg	OD

In the following days, the patient remained stable, with clinical progress closely monitored through repeat neck and chest CT scans. These scans revealed improvement in the neck and floor of the oral cavity. However, due to the patient's persistent fever and widespread infection, the maxillofacial team decided to perform a further surgical extraction of 10 teeth and continued administering IV antibiotics.

The patient underwent a chest X-ray, followed by an assessment from the thoracic team after the initial thoracotomy. Based on the X-ray findings and the clinical presentation, a second thoracotomy was performed. During this procedure, the cardiothoracic (CTC) team drained a significant amount of pus from the area of the main interlobular fissure and recommended regular flushes. They placed three drainage tubes on the right side, which led to clinical improvement as reactive fluid was observed in the drains. Despite the progress, the patient remained pyrexic with a temperature of 38.7°C. The right-side drains produced 100 mL of serous fluid over 24 hours, while the left-side drains showed no fluid output.

In the following days, the patient remained stable, and the blood results improved (Table 8). A neck and thorax CT scan revealed reduced collections in the right parapharyngeal, submandibular, and submental areas, along with decreased entrapped pleural effusions. After further assessment, the maxillofacial surgery and ENT teams concluded that no additional intervention was necessary, and oral drains could be removed. The CTC team also recommended removing one chest drain on the right side.

**Table 8:** Blood tests on day 23. The results show marked improvement in infection and inflammation markers, with both kidney and liver function showing significant progress, approaching normal ranges. WBC, white blood cells; NEU, neutrophils; CREA, creatinine; CRP, C-reactive protein; ALB, albumin; T.Bil, total bilirubin; LDH, lactate dehydrogenase; CPK, creatine phosphokinase; AST, aspartate transferase; ALT, alanine transaminase; U&E, urea and electrolyte; LFTs, liver function tests; PCT, procalcitonin; INR, international normalized ratio; FBC, full blood count

FBC			U&E			Inflammation Marker and Clotting			LFTs		
WBC (×10^9^/L)	12.7	4.0-11.0	Urea (mmol/L)	5.5	2.5-7.8	CRP (mg/L)	3.6	<2	ALB (g/dL)	2.2	3.5-5.2
NEU (%)	78	20-75	CREA (μmol/L)	72	70-150	INR	1.38	0.8-1.2	T.Bil (mg/dL)	1.8	0.2-1.3
									LDH (U/L)	687	122-222
									CPK (mcg/L)	50	10-120
									AST (U/L)	53	8-45
									ALT (U/L)	18	7-56
									PCT (ng/mL)	0.8	<0.05

**Figure 6:**
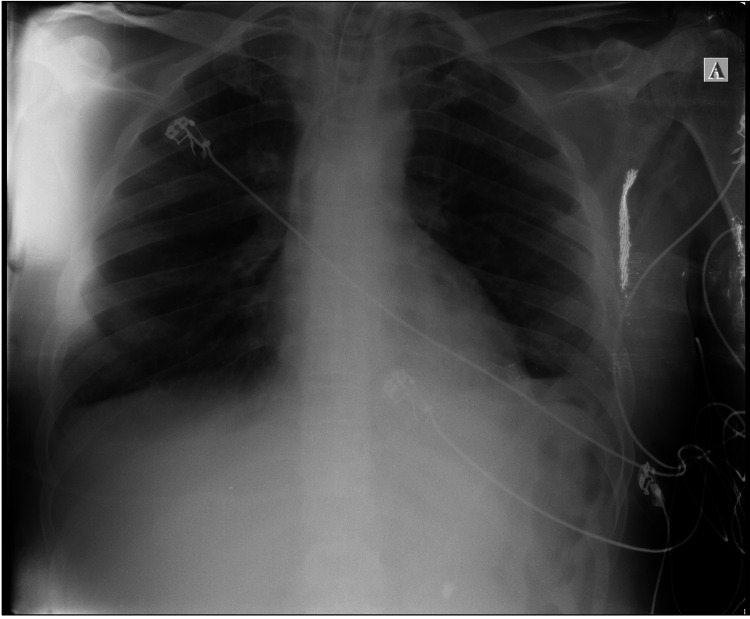
Final X-ray taken before discharge. The pleural effusion seen in the previous X-ray has resolved.

In the following days, the patient's clinical condition gradually improved, allowing for the weaning of oxygen. On day 32, a surgical tracheostomy was performed, followed by continued oxygen weaning per the patient's clinical progress. By day 38, all chest tubes and drains were removed. After approximately 40 days of hospitalization, the patient was discharged from the ICU in a hemodynamically stable condition, without fever, and with improved imaging findings. The final chest X-ray taken post-ICU is shown in Figure 6.

## Discussion

Descending necrotizing mediastinitis (DNM) is a rare, life-threatening condition characterized by the rapid spread of infection within the mediastinum, originating from head or neck infections [1]. DNM remains an uncommon but critical entity with a mortality rate of 40%, although this has decreased in recent years, highlighting the enhancements in imaging and antibiotics [2,3]. The fulminant progress of disease rapidly leads to sepsis and multiorgan failure [4].

The most common source of DNM is odontogenic infections [8]. In our patient, the source of the infection was identified as the right second lower molar, position 47. Interestingly, Reuter et al. noted that 43.1% of the patients were previously treated with a single antibiotic due to a pre-existing infection, but the polymicrobial nature of DNM required adjustments to treatment [1]. Inadequately treated head and neck infections frequently disseminate to the mediastinum via deep fascial planes [4]. The spread of infection is mediated by gravity, respiration, the negative intrathoracic pressure in the mediastinum and pleural cavities during inspiration, and the absence of barriers in the fascial planes [9,10]. To sufficiently understand the pathways of spread, complications, and surgical treatment of DNM, a thorough understanding of the anatomy of the cervico-mediastinal fascial planes and spaces is required [11,12].

The presence of risk factors impacts patients with DNM, as they are predisposed to the increased severity and frequency of disease. Extensive reviews found that a large percentage of patients suffer from pre-existing diseases. However, young, healthy patients with no significant comorbidities were diagnosed with DNM [13]. In our case, the past medical history was nonsignificant, with risk factors identified as poor dental hygiene and chronic nicotine use.

The diagnosis of DNM demands a high index of suspicion, especially in patients with a history of preceding head and neck infections. Early diagnosis, facilitated by CT imaging, is the gold standard for diagnosing DNM and is crucial for swift treatment response. Multi-planar CT is indispensable in understanding the complex anatomy of the deep neck spaces for diagnosis [14]. In our case, the neck CT was vital for identifying the foci of infection. The establishment of characteristic radiological findings is a key diagnostic criterion for DNM. Scaglione et al. identified diagnostic features of descending cervical mediastinitis on CT scanning, as detailed in Table 9 [7].

**Table 9:** Diagnostic features of descending necrotizing mediastinitis on CT [7] CT: computed tomography

Diagnostic features of descending cervical mediastinitis on CT scanning
Increased density of the adipose tissues
Vascular thrombosis
Mediastinal fluid collection
Cervical lymphadenopathy
Myositis
Pleural and/or pericardial fluid collections

Laboratory studies, including blood cultures and inflammatory markers, aid in confirming the diagnosis and guiding antimicrobial therapy. A vital consideration in the overall management of DNM is the identification of the causative microorganisms. Kappus and King estimate that approximately 58% of DNM patients suffer from polymicrobial infection [4]. The most frequently isolated microorganisms are streptococci and anaerobic bacteria, native to the odontogenic or pharyngeal nature [9]. As discussed by Kocher et al., the administration of IV broad-spectrum antibiotics with coverage for aerobic and anaerobic bacteria as soon as possible is mandatory regarding the high mortality rates of up to 85% in the pre-antibiotic era [15]. However, as evident in our case, antibiotic use without drainage of the infection foci hinders treatment success and delays clinical improvement.

Currently, the surgical approach to treat DNM remains controversial. A large body of evidence supports the approach of early concomitant transcervical and transthoracic (posterolateral thoracotomy or video-assisted thoracoscopic surgery {VATS}) surgical interventions for the management of DNM [12]. As Kocher et al. note, the surgical approach is guided by the extension of disease in the upper or lower part of the mediastinum [15]. The approach must consider the severity of the illness, the patient's condition and comorbidities, and the surgeon's experience level [15].

The cornerstones of the management of DNM are early escalation, aggressive medical and surgical management, and a multidisciplinary approach [9]. In the absence of official guidelines, the consensus of treatment is prompt airway management, the administration of broad-spectrum antibiotics, and surgical intervention to eliminate infective foci of the neck and mediastinum. Finally, the involvement of specialities such as microbiology, thoracic surgery, intensive care, anesthesiology, and otorhinolaryngology with a shared-care plan is vital in reducing mortality.

## Conclusions

Descending necrotizing mediastinitis (DNM) is a severe form of mediastinitis, posing significant challenges to clinicians due to its aggressive nature and potential for rapid deterioration. Immediate and aggressive intervention, often involving surgical drainage and broad-spectrum antimicrobial therapy, is essential to mitigate the progression of the disease and improve outcomes. This case report highlights the importance of the heightened clinical suspicion of DNM in patients with head and neck infections that do not improve despite broad-spectrum antibiotics. It showcases the importance of communication and effective teamwork within the multidisciplinary team in managing this condition effectively.
